# The effect of resistin on the redox state of breast cancer cells

**DOI:** 10.1007/s00432-023-05556-7

**Published:** 2024-01-22

**Authors:** Elitsa Pavlova, Radoslav Stojchevski, Dimiter Avtanski

**Affiliations:** 1 Faculty of Physics, Sofia University “St. Kliment Ohridski”, 5 James Boucher Blvd., 1164 Sofia, Bulgaria; 2 Friedman Diabetes Institute, Lenox Hill Hospital, Northwell Health, New York, NY 10022 USA; 3 Donald and Barbara Zucker School of Medicine at Hofstra, Northwell, Hempstead, NY 11549 USA; 4 Feinstein Institutes for Medical Research, Manhasset, NY, 11030 USA

**Keywords:** Oxidative stress, Antioxidant activity, Resistin, Leptin, Vitamin C, Breast cancer

## Abstract

**Purpose:**

Resistin is an inflammatory cytokine secreted mostly by adipocytes and immune cells that plays a role in the development of insulin resistance, diabetes, and cancer. We hypothesized that resistin’s inflammatory activity influences the free radical and oxidative stress pathways.

**Methods:**

We used human breast carcinogenic (MCF-7 and MDA-MB-231) and non-carcinogenic (MCF-10A) cells in this investigation and correlated the absorbed resistin concentration with the change in oxidative stress (TBARS, carbonated proteins) and antioxidant activity (Antioxidant Capacity, SuperOxideDismutase, CATalase, Glutathione Peroxidase).

**Results:**

Resistin was substantially more effective as a prooxidant at lower (12.5 ng/ml) concentrations, than at higher concentrations (25.0 ng/ml). Vitamin C did not appear to be an effective oxidative stress protector at antioxidant concentrations of 5.10^–4^ M. Leptin, at 100 ng/ml, did not result in conclusive oxidative stress or antioxidant defence stimulation, as expected.

**Conclusion:**

Taken together, the findings support resistin’s role as a non-oxidative stress marker and a metabolic signaling molecule.

## Introduction

Breast cancer is the most common cancer in women, and metastasis is the primary cause of death. A critical factor in cancer metastasis is the epithelial-to-mesenchymal transition (EMT) that transforms the cancer cells into mesenchymal ones. EMT leads to resistance to tumor treatment and worsens the anticancer immune response.

Resistin is an inflammatory cytokine that is secreted mostly by adipocytes and immune cells. Its production is directly connected to insulin resistance and diabetes (WHO 2023). Our previous research (Avtanski et al. 2019) and other studies (Dalamaga 2014; Dasgupta and Klein 2014; Wang et al. 2018) have demonstrated that resistin induces EMT, thereby promoting breast cancer metastasis and progression.

The body`s natural oxygen metabolism involves free radical processes, including toxic product exchange, cell membrane renewal, immune response, and cellular signaling (Sies 2019; Sharifi-Rad et al. 2020; Pavlova and Savov 2006; Pavlova et al. 2016). Reactive compounds, including free radical products and reactive oxygen species (ROS), are generated (Pizzino et al. 2017). In a healthy state, antioxidant enzymes and other antioxidant substances balance the generation of free radicals and ROS. However, inflammation disrupts this equilibrium, favoring oxidative processes (Sies 2019; Sharifi-Rad et al. 2020). This discrepancy between the generation of free radicals and ROS and the body’s capability to neutralize their detrimental effects through detoxification is referred to as oxidative stress.

Lipid peroxidation is a crucial component of the oxidative stress process. It is a major indicator of oxidative stress in the organism. The decomposition of lipid peroxides results in the formation of many compounds, including reactive carbonyl compounds (e.g., MDA), which are usually measured as TBARS (thiobarbituric acid reactive substances) (Pizzino et al. 2017; Sies 2019).

Protein carbonization is another post-translational modification that occurs during oxidative stress, resulting in the generation of protein carbonyl adducts. Carbonyl groups are generated at various amino acids by radical and non-radical ROS (Dalle-Donne et al. 2003).

In cellular and extracellular environments, significant amounts of superoxide dismutase (SOD) are crucial in the prevention of diseases associated with oxidative stress. SOD is critical in preventing neurodegenerative and many other disorders (Sharifi-Rad et al. 2020).

Catalase (CAT) is an omnipresent antioxidant enzyme in aerobic cells. CAT detoxifies hydrogen peroxide (H_2_O_2_), a toxic byproduct of aerobic metabolism and pathogenic ROS generation. CAT also protects cancer cells against the cytotoxic effects of H_2_O_2_ (Klingelhoeffer et al. 2012; Sharifi-Rad et al. 2020).

Glutathione peroxidase (GPX) is a group of enzymes with peroxidase activity. They are major players in protecting against oxidative damage (Muthukumar and Nachiappan 2010).

The total antioxidant capacity (AOC) is a parameter that may give more valuable biological information than measuring single components because it evaluates the total, even synergistic effects, of all the antioxidants presented.

In a healthy organism, the two groups of biomarkers (pro- and antioxidant) are usually inversely correlated: when prooxidant markers (such as TBARS and carbonated proteins (CP)) increase, the antioxidant indicators (such as AOC, SOD, CAT, and GPX) tend to decrease, and vice versa (Sies 2019; Sharifi-Rad et al. 2020). However, in certain cases, such as cancer diseases, the initial line of resistance against ROS and oxidative stress is to stimulate the antioxidant defense mechanisms with higher activity and capacity, even before the accumulation of final and stable prooxidant products (Hayes et al. 2020; Griñan-Lison et al. 2021). The direct dependence of oxidative stress and ROS generation on cancer progression is supported by many pieces of evidence (Gorrini et al. 2013; Athreya and Xavier 2017; Hayes et al. 2020; Griñan-Lison et al. 2021; Hadi et al. 2021). Cells can adapt to oxidative stress through metabolic and genetic reprogramming on a larger scale. Compared to normal cells, tumor cells present high ROS concentrations and strict regulation of the redox balance to sustain low levels of oxidative stress. In the cases of breast cancer, different cell subtypes are observed presenting various redox statuses (Griñan-Lison et al. 2021).

The influence of oxidative stress on the metastatic process is conflicting, probably due to various redox limitations controlling the pathways for cell division, mobility, and survival rate. All these depend on the types of ROS, affected subcellular compartments, as well as the antioxidant transcription factors, and the frequency of switching from epithelial or mesenchymal phenotypes. The metastatic tumor cells easily adapt to the new conditions in a specific tissue, which is controlled by “phenotypic stability factors” (e.g., grainy head-like 2 (GRHL2), ovo-like zinc finger 2 (OVOL2), DNp63a, NUMB, etc.) that balance such hybrid phenotypes. The premalignant cells need to adapt to the high concentrations of ROS induced by the activated oncogenes. That causes additional requirements on the antioxidant system too (Dasgupta and Klein 2014; Hadi et al. 2021).

Based on the aforementioned research, we hypothesized that resistin affects inflammation through a free radical and an oxidative stress mechanism. To test this, we used human breast carcinogenic (MCF-7 and MDA-MB-231) and non-carcinogenic (MCF-10A) cells, and correlated the amount of absorbed resistin content in the cells with their oxidative stress status and antioxidant capacity.

## Materials and methods

### Cell cultures

Human breast cancer MCF-7 (Cat. # HTB-22) and MDA-MB-231 (Cat. # CRM-HTB-26), and non-carcinogenic MCF-10A (Cat. # CRL-10317) breast epithelial cells were delivered from the American Type Culture Collection (ATCC). The cells were cultivated at 37 °C and 5% CO_2_ in either Dulbecco’s Modified Eagle’s Medium (DMEM) (Corning, Inc., Cat. # 10-013-CF) with 10% FBS (VWR International, Cat. # 1500-500) and 1 × Antibiotic/Antimycotic Solution (Corning, Inc., Cat # 30-004-CL) (MCF-7 and MDA-MB-231 cells) or HuMEC Ready Medium kit (Cat. # 12-752-010, Gibco, Thermo Fisher Scientific) (with HuMEC Basal Serum-Free Medium, HuMEC Supplement, and Bovine Pituitary Extract (BPE)) and 1 × Antibiotic/Antimycotic Solution (MCF-10A cells).

The tested lysates are prepared after collecting the cells by centrifugation at 2000 g for 10 min at 4 °C, earlier detached by trypsine. Afterward, the cells are homogenized on ice in 2 ml buffer and centrifuged at 10,000 g for 15 min at 4 °C. The supernatant was frozen at – 80 °C and later used to test the chosen biomarkers.

### Experimental design

For the experiments, 2.2 × 10^6^ cells were seeded in 6-well plates. The following day, the cells were treated with resistin (EMD Millipore Corp., Cat. # PF-138) (12.5 or 25 ng/ml for 24, 48, and 72 h). Ascorbic acid (vitamin C) (Sopharma, Bulgaria, Cat. # 10018989) was used as a standard antioxidant in a concentration of 5 × 10^–4^ mol/l, and leptin was used as a prooxidant (BioVision, Cat. # 4366-02) in a concentration of 100 ng/ml. They were added instead of resistin to the plates. The standard “blank” control was applied in each experiment—cells without any affecting substance added.

### Oxidative stress markers measurements

TBARS, carbonyl content measurements, SOD, CAT, GPX activities, and AOC were measured using kits from Cayman Chemicals (Cat. ## 10009055, 10005020, 706002, 707002, 703202, and 709001), following the manufacturer’s protocols. Detailed information on each method and its protocol can be found at https://www.caymanchem.com/products/categories.

### Statistical analyses

All results were received as triple-reproducible measurements. Statistical calculations (Student’s *t* test) were processed by Origin 8.5 (OriginLab) and Microsoft Office Excel 2010 (Microsoft Corp.) software. Data were accepted as statistically significant when *p* ≤ 0.005.

## Results and discussion

Our research findings indicate that resistin plays a role as both a non-oxidative stress marker and a metabolic signaling molecule. Lower concentration of resistin was found to be more effective in eliciting a prooxidant response.

Our study found evidence contradicting the hypothesis that resistin affects inflammation through a direct free radical and an oxidative stress mechanism. The results indicated a low correlation between the amount of resistin absorbed by the cells, and the levels of oxidative stress and antioxidant capacity.

TBARS and CP are indicators of oxidative damage in the cells. We hypothesized that resistin and leptin would increase the levels of those redox markers (Morawietz and Bornstein 2006; Dong et al. 2006).

Short-living ROS that are very challenging to measure directly are usually detected through the measurement of TBARS, the final products of lipid peroxidation. TBARS and CP are chemically and metabolically stable, making them useful targets for detecting oxidative damage. Protein carbonization can also be catalyzed by redox cycling cations such as Fe^2+^ or Cu^2+^, in conjunction with non-radical ROS, toxicants, or by secondary reactions with aldehydes produced during lipid peroxidation (Dalle-Donne et al. 2003).

### Prooxidant biomarkers (Fig. 1)

**Fig.1:**
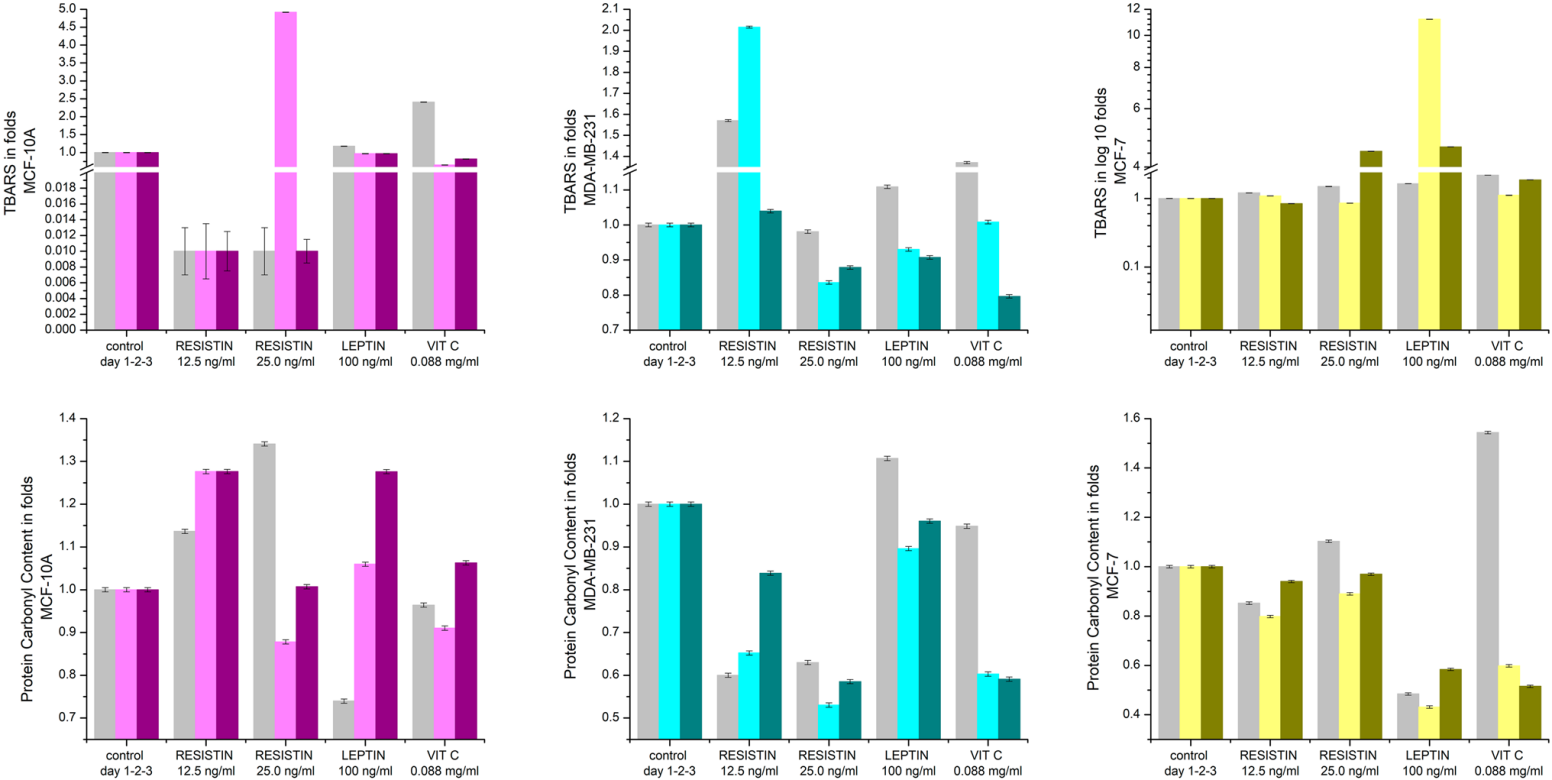
The effects of resistin, vitamin C, and leptin on the level of TBARS (**A**) and carbonate proteins (CP) (**B**), presented in folds (*p* ≤ 0.005)

#### MCF-10A cells

Leptin (100 ng/ml) did not affect TBARS concentrations on days 1, 2, and 3 of treatment, while resistin (12.5 ng/ml) even suppressed the accumulation of those products. At concentrations of 25.0 ng/ml, resistin stimulated the generation of TBARS almost 5 times on day 2 of treatment, but it showed inhibitory effects on days 1 and 3.

Vitamin C (0.088 mg/ml) reduced TBARS by up to 40% on days 2 and 3 while increasing the expression of those products by 2.4-fold on day 1 of treatment.

CP concentrations were affected by both resistin and leptin: resistin, especially at the lower concentration, increased the level of CP up to 30%; leptin did so up to 30% on the 3rd day of treatment.

Vitamin C treatment did not provoke any significant change in CP concentrations.

#### MDA-MB-231 cells

Resistin (12.5 ng/ml) caused up to a two-fold accumulation of TBARS on days 1 and 2, but the effect was lost on day 3. Resistin (25.0 ng/ml) slightly suppressed the generation of TBARS on all treatment days. A similar effect was observed with leptin, except on day 1 of treatment, when a slight increase was detected. Vitamin C stimulated the accumulation of TBARS (up to 40%) only on day 1, while it was entirely absorbed on days 2 and 3.

CP content was significantly inhibited (20–50%) on all treatment days with both applied resistin concentrations. Leptin slightly increased the concentration of CP.

Vitamin C decreased CP levels by up to 40% on day 2 of treatment, and the effect was maintained on day 3.

#### MCF-7 cells

Resistin (12.5 ng/ml) did not affect the accumulation of TBARS. Resistin (25.0 ng/ml) caused the accumulation of those products more than 4 times compared to the control or resistin (12.5 ng/ml) on day 3. Leptin was effective and increased TBARS levels by more than 11 times on day 2 and almost 5 times on day 3 of treatment.

Vitamin C showed weak prooxidant effects on TBARS concentrations on days 1 and 3.

CP levels were diminished by 5–20% after treatment with resistin and slightly increased on day 1 after treatment with resistin (25.0 ng/ml). The results were similar to MDA-MB-231. Leptin inhibited the CP content by up to 50% on all treatment days.

Vitamin C acted as a prooxidant on day 1 (50%) and as an antioxidant (50%) on days 2 and 3 post-treatment.

AOC and the enzymes superoxide dismutase (SOD), catalase (CAT), and GPX are important indicators of cellular recovery and defense. In healthy cells, these components are usually suppressed or exhausted upon damage or oxidative stress.

The total antioxidant capacity of the extracellular fluid is the sum of endogenous and food-derived antioxidants. Therefore, measuring the overall AOC may ensure more pertinent biological data than measuring single concentrations and activities, as it sums up the effects of all the presented antioxidants in the blood plasma and other fluids of the body (Rice-Evans 2000; Proteggente et al. 2002; Pavlova and Savov 2006; Pizzino et al. 2017; Sies 2019; Sharifi-Rad et al. 2020).

SOD catalyzes a rapid turnover of the superoxide radicals (2 × 10^6^ M^−1^ s^−1^), maintaining low levels of these radicals in cells and tissues. Therefore, measuring SOD activity is significant in characterizing the antioxidant abilities of a biosystem (Sies 2019; Sharifi-Rad et al. 2020).

The catalytic activity of CAT is described by the conversion of two molecules of hydrogen peroxide into a molecule of oxygen and two molecules of water. Its peroxidase activity is described when low-molecular-weight alcohols can act as electron donors. Other enzymes with peroxidase activity do not use the aliphatic alcohols that CAT employs as a specific substrate (Sies 2019; Sharifi-Rad et al. 2020).

GPX converts lipid hydroperoxides into alcohols and free hydrogen peroxide into water (Muthukumar et al. 2011). Glutathione reductase is a flavo-enzyme that reduces glutathione (GSSG) to glutathione (GSH) by NADPH, maintaining adequate concentrations of GSH in the cells (Sies 2019; Sharifi-Rad et al. 2020). Combining GSH inhibitors with various anticancer supplements may appear to be another approach to exterminating cancer cells (Gorrini et al. 2013).

### Antioxidant biomarkers (Fig. 2)

**Fig.2:**
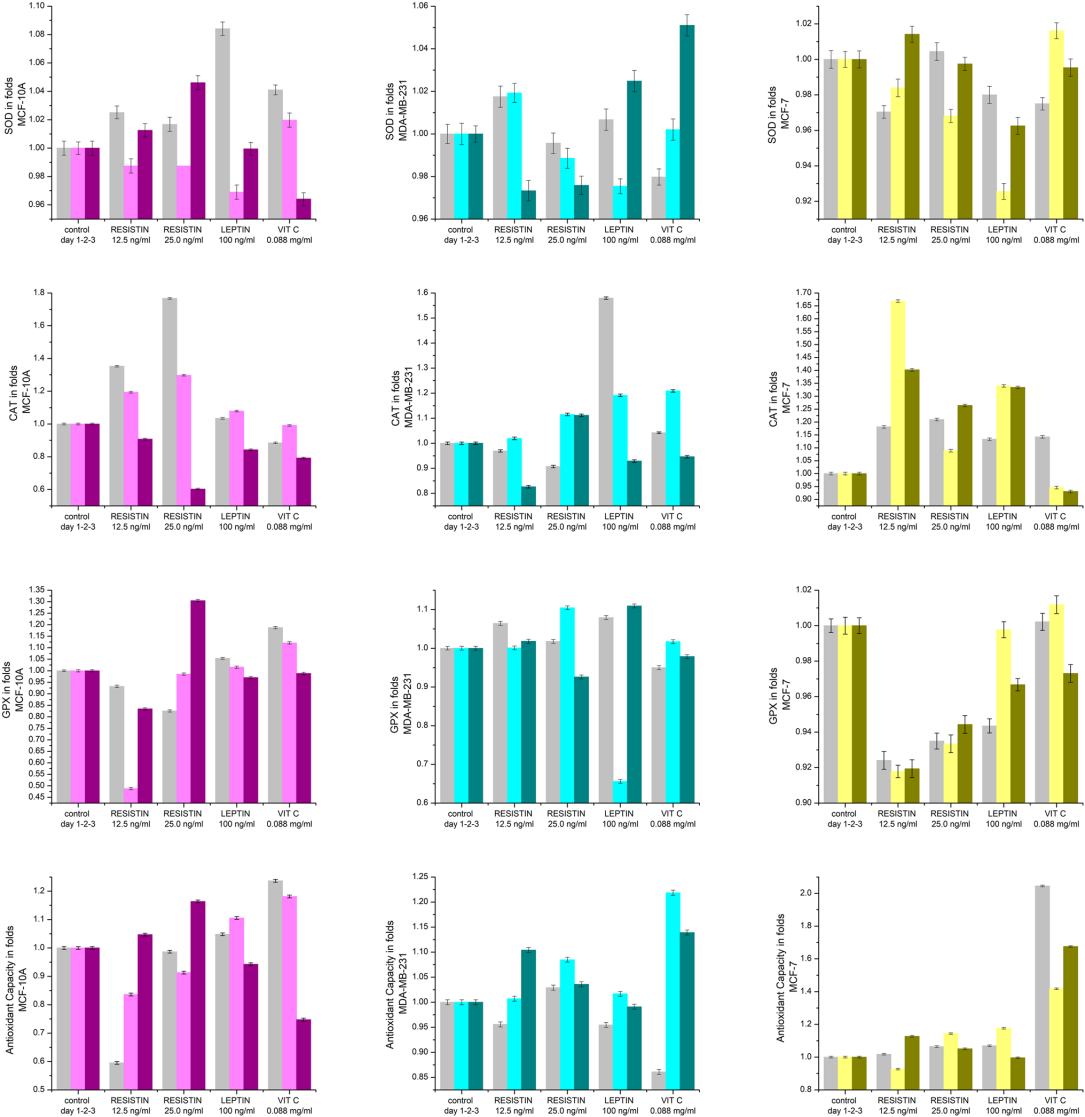
The effects of resistin, vitamin C, and leptin on the level of SOD (**A**), CAT (**B**), GPX (**C**), and AOC (**D**) presented in folds (*p* ≤ 0.005)

#### MCF-10A cells

In those cells, resistin (12.5 ng/ml) suppressed AOC by up to 40% on day 1 and 20% on day 2, but kept control levels on day 3 post-treatment. Resistin (25.0 ng/ml) stimulated AOC levels on day 3 by less than 20%. Leptin slightly suppressed that marker on day 3.

Vitamin C contributed to the increase in AOC (20%) on days 1 and 2 of the treatment but had a suppressive effect on day 3.

Resistin, leptin, and vitamin C did not significantly affect SOD activity in MCF-10A cells.

CAT levels were upregulated by both resistin concentrations on days 1 and 2 (20–80%) but suppressed on day 3. Leptin had no effects in the first two days of treatment but suppressed CAT activity by 20% on day 3.

Vitamin C did not show any protective or stimulating effect on CAT activity.

Resistin (12.5 ng/ml) inhibited the activity of GPX by 50% on day 2 of the treatment, but the observed effects on days 1 and 3 were light. Resistin (25.0 ng/ml) stimulated GPX activity by 30% after 3 days of treatment. Leptin showed no significant effect on GPX activity.

Vitamin C was most potent on day 1 post-treatment; however, that effect was transient.

#### MDA-MB-231 cells

Resistin and leptin did not affect the AOC in that cell line.

Vitamin C stimulated the total antioxidant activity on days 2 and 3 (~ 20%).

Resistin, leptin and vitamin C had no effect on SOD activity in that cell line.

CAT was affected by resistin and leptin: resistin (12.5 ng/ml) inhibited its activity by 20% on day 3 of treatment, while leptin stimulated CAT activity mostly on day 1 (50%).

Vitamin C contributed to CAT activity mostly on day 2–20%.

Resistin did not significantly affect the activity of GPX. Leptin inhibited GPX activity on day 2 by 35%.

Vitamin C had no significant effect on GPX activity.

#### MCF-7 cells

Resistin and leptin had no substantial effect on AOC in that cell line either.

Vitamin C showed a strong contributing effect to AOC in MCF-7: with 40–100% higher total antioxidant activity.

Resistin slightly diminished the activity of SOD, while leptin had a stronger effect, especially on day 2.

Vitamin C did not show up as an antioxidant contributor there.

Resistin presented a definitive stimulating activity on CAT in that cell line. Its effect was much higher in the lower concentration (almost 70%). Leptin also stimulated CAT activity (30% on days 2 and 3).

Vitamin C had no significant effect on CAT activity in that cell line.

GPX was slightly more inhibited by resistin in comparison to leptin in MCF-7.

Vitamin C also had no significant effect on GPX activity in that cell line at the chosen concentration.

In general, the hyper-proliferation of tumor cells is accompanied by the hyperproduction of ROS. For these reasons, tumor cells fight to improve their antioxidant status. On the other side, they avoid ROS concentrations that would trigger apoptosis, ferroptosis, or pathology (Redza-Dutordoir and Averill-Bates 2016; Dodson et al. 2019).

Vitamin C plays many roles in the organism: a reducing agent; an antioxidant and free radical scavenger; and a prooxidant in higher concentrations. It is important for mitosis and the healing and repair mechanisms in the tissues. In concentrations such as 20 mM, it induces the death of the cancerous cells (Hadi et al. 2021). It is well established that vitamin C plays the role of an oxidizing or reducing agent, depending on the applied concentration. Usually, lower doses of vitamin C present good antioxidant properties and prevent oxidant-induced apoptosis; higher doses of vitamin C usually induce pro-oxidation and apoptosis. In fact, 0.3–20 mM concentrations of vitamin C induce the production of H_2_O_2_ and ascorbate radicals, which eliminate cancerous cells (Hadi et al. 2021). In our study, we applied vitamin C as an antioxidant in concentrations of 5.10^–4^ M.

Another reference that we have applied is leptin. It is expected to provoke the generation of free radicals and ROS. It was applied as a prooxidant. It has been observed that higher leptin levels induce the generation of ROS, mostly due to the activation of NADP-oxidase (Dong et al. 2006; Morawietz and Bornstein 2006). It has also been observed that the replacement leptin therapy significantly downregulated the expression of NADPH oxidase (Frühbeck et al. 2017). On the other side, when leptin is added at 0.1 and 1 mg/ml concentrations, it strongly decreases the EPR signal due to its scavenging activity toward O_2_^•−^ radicals (Macrea et al. 2013). This represents leptin`s protective role at physiological concentrations. We have applied leptin in 100 ng/ml concentrations, which are extremely low and have no or very light antioxidant activity.

In breast cancer, oxidative stress usually causes DNA damage, changing the transcription and signal transduction processes that result in errors in replication, genomic instability, and the activation of carcinogens. There are a few risk factors in the progression of breast cancer that are linked to the generation of ROS. These are genetic predisposition, aging, menopause, and the level of estrogens, which lead to DNA damage and chromosomal aberrations. All these are responsible for the development and progression of breast cancer (Klingelhoeffer et al. 2012; Dasgupta and Klein 2014). The human cancer cell lines that we have tested demonstrated considerable differences in their resistance to resistin-provoked oxidative stress.

The free radicals and ROS in the cancerous cells stimulate proliferation and cell survival by suppressing various activities (PTPs, PTEN, MAPK phosphatases, MAPK–ERK, PI3K–PKB/Akt, and PKD–NF-kB signaling cascades). The tumor cells control the antioxidant transcription factors and reprogram the metabolism to increase NADPH and the de novo synthesis of GSH, utilizing and balancing the proliferative benefits of the higher ROS concentrations on one side, and mitigating the risk of damage and death on the other (Hayes et al. 2020).

The tumor cells may activate anti-apoptotic pathways. For example, breast cancer cells employ the TRPA1 channel and activate Ca^2+^ signaling, ERK and PI3K–PKB/Akt pathways, and activate MCL-1, which is responsible for tolerance to oxidative stress and drug metabolism. NRF2 is responsible for the expression of TRPA1. It strengthens the connection between redox homeostasis and Ca^2+^ signaling and extends the influence of NRF2. Other proteins that play a role in Ca^2+^ homeostasis are sensitive to S-glutathionylation too (Hayes et al. 2020).

MDA-MB-231 breast cancer cells usually produce much higher concentrations of H_2_O_2_ in comparison to MCF-7 breast cancer cells. The results from ROS on the EMT process probably vary due to the cancer type and/or EMT-dependent differences. Those variations present heterogeneity in tumor cells due to reasons controlling the toggle between epithelial and mesenchymal cell types (Hayes et al. 2020).

There is controversial information about the effects of various antioxidants in the treatment of cancer, especially breast cancer. One and the same substance may present as a prooxidant or an antioxidant depending on its concentration, cell type, treatment time, etc. For example, investigations about the effects of vitamin C in rats and at the same doses in humans show that vitamin C forms ascorbate and H_2_O_2_ in the extracellular fluid, but this does not happen in the blood (Dasgupta and Klein 2014; Griñan-Lison et al. 2021).

A meta-analysis of breast cancer suggested that the application of vitamin C as an antioxidant in the treatment of cancer may significantly reduce the risk of patient mortality. In fact, vitamin C plays the role of a selective prooxidant against some types of tumor cells, like breast cancer. Vitamin C causes ROS generation and glutathione oxidation (Griñan-Lison et al. 2021).

Not all tumor types are sensitive to ROS; for these reasons, one can expect no benefits from various antioxidants in those cases (Athreya and Xavier 2017).

Many antioxidants show no efficacy due to endogenous factors. They may be unable to decrease the ROS levels and the lipid peroxidation process and protect the cells from oxidation. It is well established that higher ROS concentrations kill cancerous cells and prevent tumor initiation and progression. For these reasons, supplementing with antioxidants at the early stages of cancer could support the survival of cancerous cells and induce tumor growth. It was proven that the intake of dietary antioxidants worsens breast cancer prognosis, enhances metastasis, and decreases the survival rate (Griñan-Lison et al. 2021).

Earlier research demonstrated that resistin induces EMT and toggles stem cell-like properties in breast cancer cells. Currently, the role of resistin in cancer progression is not well understood, and the molecular mechanisms of resistin signaling are largely unknown. Establishing a mechanism by which resistin promotes EMT in breast cancer cells could reveal potential molecular targets for the development of novel diagnostic tools and treatments for breast cancer (Pavlova et al. 2016; Pizzino et al. 2017).

## Conclusion

The obtained results present the role of resistin as a non-oxidative stress and a metabolic signaling molecule. The lower concentration of resistin (12.5 ng/ml, compared to 25.0 ng/ml) is much more effective in its prooxidant response. The obtained data present the results of two carcinogenic breast cancer cell lines compared to a non-carcinogenic one. Obviously, the major signaling effect of resistin in the organism is not through the free-radical oxidative mechanism.

When applied at concentrations (5.10^–4^ mol), Vitamin C does not appear to be an effective protector on all the tested oxidative stress biomarkers and cell lines. When applied at 100 ng/ml, leptin does not provoke definitive oxidative stress or stimulation of the antioxidant defense.

The knowledge obtained from this research would help to better understand the signaling mechanisms behind how resistin promotes inflammation in breast cancer cells.

## Data Availability

The data that support the findings of this study are available from the corresponding author upon reasonable request.
